# Population proteomics for equitable precision medicine

**DOI:** 10.1038/s44321-026-00508-3

**Published:** 2026-08-14

**Authors:** Xiaobo Yu, Jing Yang, Bomiao Yu, Jin-tai Yu

**Affiliations:** 1 State Key Laboratory of Medical Proteomics, Beijing Proteome Research Center, National Center for Protein Sciences-Beijing, Beijing, China; 2 Guangzhou National Laboratory, Guangzhou International Bio Island, Guangzhou, China; 3 International Academy of Phronesis Medicine, Guangzhou, China; 4 The π-HuB Project Infrastructure, Guangzhou, China; 5 Department of Neurology and Institute of Neurology, Huashan Hospital, State Key Laboratory of Medical Neurobiology and MOE Frontier Center for Brain Science, Shanghai Medical College, Fudan University, Shanghai, China

**Keywords:** Proteomics

## Abstract

This Comment discusses how recent advances are moving population proteomics beyond biomarker discovery toward equitable clinical translation through cross-population validation, mechanistic multiomics and global research infrastructures.

**Figure:**
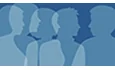

## Teaser

Population proteomics is emerging as a new framework for equitable precision medicine. By studying protein variation across populations, this field bridges population genomics and conventional proteomics to capture the functional molecular states through which genetic ancestry, environmental exposures and other contextual factors shape human health. Here, we discuss how recent advances are moving population proteomics beyond biomarker discovery toward equitable clinical translation through cross-population validation, mechanistic multiomics and global research infrastructures. Its ultimate promise is not to classify populations as fixed biological categories, but to make human diversity measurable, interpretable and clinically actionable for equitable precision medicine.

## Population proteomics comes of age

**Figure 1:**
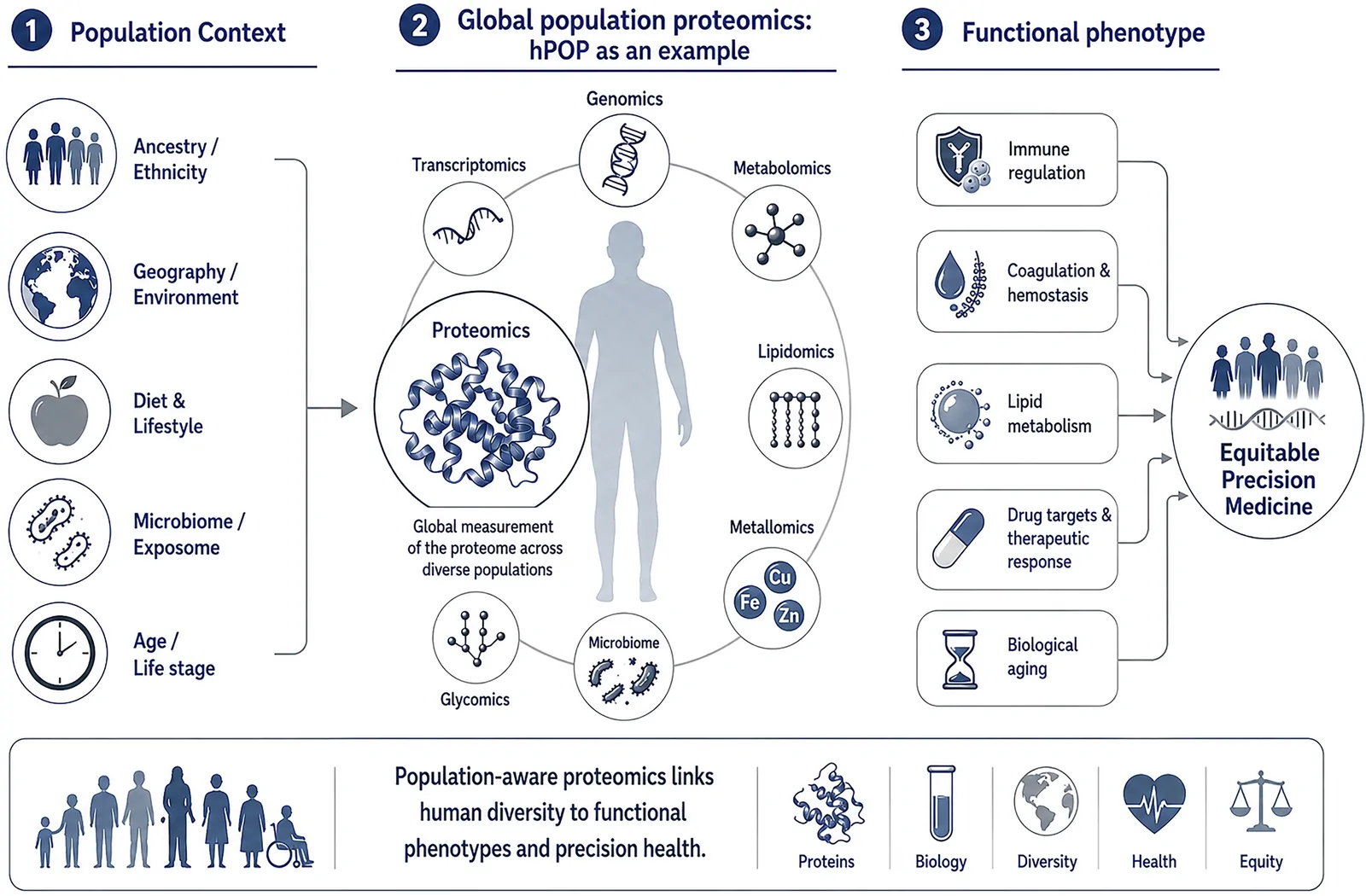
Population proteomics from single-region discovery to global precision medicine. Early population proteomics has been shaped by large single-region or ancestry-skewed resources that link circulating proteins to genetics, health traits, diseases, and drug targets. The next phase requires cross-region and multi-population designs that explicitly model ancestry, geography, diet, microbiome ecology, age, and environmental exposure. hPOP provides a proof-of-principle case for this transition, whereas larger initiatives such as π-HuB can extend this framework into population-aware reference maps, longitudinal disease trajectories, biomarker recalibration, drug–target prioritization, and therapeutic stratification for equitable precision medicine.

Population proteomics is the systematic study of protein variation across human populations to understand how genetic ancestry, environmental exposures, and life contexts shape molecular phenotypes and influence disease risk and therapeutic response. The field has evolved through three major phases: systematic proteome mapping established the foundation for comprehensive protein characterization; large-scale cohort-based proteomics connected molecular variation with genetics, health, and disease; and emerging global population proteomics initiatives are integrating multi-country, multi-ancestry, and multiomic datasets to decode how human diversity is represented at the molecular level, ultimately enabling equitable precision medicine (Aebersold and Mann, 2016; Deng et al, 2025; Barapour et al, 2026) (Fig. 1).

Genomics has transformed the study of inherited variation, although genetic discovery remains strongly biased toward populations of European ancestry, limiting the global transferability of precision medicine (Sirugo et al, 2019). Even when applied at the population scale, genomics primarily captures inherited genetic variation, yet DNA sequence alone cannot explain how age, diet, geography, infection, microbiome ecology, social environment, and treatment exposure combine to shape disease-relevant biology. Proteins, by contrast, provide a functional readout of these influences. They execute cellular functions, mediate inflammatory and metabolic pathways, constitute the targets of most drugs, and often serve as clinically measurable biomarkers. Protein measurements, therefore, provide a more direct link between genetic architecture, environmental exposures, and physiology than DNA sequence alone. Conventional proteomics has often focused on discovery in small cohorts or disease-specific comparisons. Population proteomics bridges genomics and conventional proteomics by integrating large-scale protein measurements with diverse population cohorts to reveal how genetic background, environmental exposures, and life experiences shape human biology, disease susceptibility, and therapeutic response.

## Why population proteomics matters for precision medicine?

The clinical value of population proteomics lies in its ability to improve the development and validation of molecular biomarkers and therapeutic targets. As large, multi-cohort studies mature, the field is moving beyond protein discovery toward clinically validated tools that support diagnosis, risk prediction, and therapeutic decision-making. The Lung Cancer Cohort Consortium, as part of INTEGRAL, analyzed prediagnostic blood samples from six prospective cohorts in the United States, Europe, Singapore, and Australia and identified 36 reproducible protein markers of imminent lung cancer diagnosis (Lung Cancer Cohort Consortium, 2023). This discovery phase was followed by validation of a protein-based INTEGRAL-Risk model for lung cancer screening eligibility, illustrating how population-scale proteomics can refine clinical decision points rather than simply generate biomarker lists (Zahed et al, 2026). In another translational example, in-depth plasma proteomics identified and validated Elafin/PI3 as a non-invasive serum biomarker for psoriasis, a chronic immune-mediated inflammatory disease affecting approximately 125 million people worldwide. Large-scale multicenter clinical validation demonstrated strong diagnostic performance, the assay was approved by China’s National Medical Products Administration (NMPA) in 2025, and Elafin/PI3 was incorporated into the 2025 expert consensus on early recognition, diagnosis, and treatment of psoriatic arthritis (Yi et al, 2025). Together, these studies illustrate how population proteomics is beginning to translate molecular discoveries into clinically useful tools for screening, prevention, diagnosis, monitoring, and therapeutic stratification.

## Towards equitable precision medicine

Clinical translation alone is not sufficient. To achieve equitable precision medicine, biomarkers and therapeutic targets must also be robust across diverse populations, geographic regions, and healthcare settings. Early population proteomics established the feasibility of linking circulating proteins with genetic variation and disease risk at scale through large biobank resources, including the UK Biobank Pharma Proteomics Project and disease-scale plasma proteome atlases (Deng et al, 2025; Barapour et al, 2026). The next challenge is determining whether protein baselines, biomarker thresholds, disease associations, and drug–target hypotheses discovered in one population remain valid across regions, ancestries, and health systems.

Cross-ancestry plasma proteome analyses in the Atherosclerosis Risk in Communities study showed that pQTLs and protein-prediction models are not automatically transferable between European American and African American participants (Zhang et al, 2022). Studies in continental African and Black American populations further reveal regulatory signals and disease-relevant protein architectures that would be underpowered or invisible in European-dominant datasets (Soremekun et al, 2026).

Cross-biobank studies reinforce this message. Cardiovascular proteomics in UK Biobank and China Kadoorie Biobank prioritized proteins with observational and genetic evidence for disease relevance (Lind et al, 2024), while proteomic risk models for ischemic heart disease and type 2 diabetes in Chinese and European adults illustrated how cross-population validation can improve prediction and nominate drug targets (Mazidi et al, 2024; Yao et al, 2024). A proteomic aging clock trained in UK Biobank and validated in China Kadoorie Biobank and FinnGen shows that protein-based biological age can be compared across geographically distinct resources, while still requiring careful calibration (Argentieri et al, 2024). Multi-cohort proteogenomic analyses, including studies across dozens of cohorts, provide powerful pQTL and drug–target maps but also reveal that sample diversity, platform harmonization, and cross-study standardization remain limiting factors (Koprulu et al, 2026). Together, these studies show that ensuring the portability and calibration of proteomic discoveries across diverse populations is becoming a defining challenge—and opportunity—for equitable precision medicine.

## hPOP as a model for population proteomics

Understanding why molecular signatures differ across populations is essential for translating population proteomics into equitable precision medicine. hPOP provides a useful model for population proteomics because it explicitly separates the contributions of ancestry, geography, and environment to molecular variation. Barapour et al profiled 322 generally healthy participants across multiple continents and integrated genomics, transcriptomics, proteomics, metabolomics, lipidomics, metallomics, glycomics, pathogen-reactive antibodies, metabolic hormones, and microbiome measurements (Barapour et al, 2026). By including European, East Asian, and South Asian ancestry groups living within and outside their ancestral regions, hPOP directly asks which molecular signals track ancestry, geography, or their interaction.

The result is a systems-level map of molecular context. hPOP identified ancestry- and geography-associated variation across metabolic, immune, microbial, and proteomic layers; highlighted pathways involving immune regulation, coagulation, lipid metabolism, and host–microbiome interactions; and showed that biological age estimates vary with ancestry and residence. Importantly, these findings do not imply that populations are fixed biological categories. Rather, they demonstrate that population-level molecular variation is shaped by genetics, environment, diet, microbiome ecology, age, and relocation, underscoring the need for precision medicine to measure these factors rather than average them away.

Within this framework, proteins provide the functional link between population-level molecular variation and clinical translation. In hPOP, differential plasma proteins included drug targets or drug-response-related proteins such as coagulation factors, immune regulators, complement components, and SHBG. The study also linked selected protein differences to population-specific regulatory variants and expression quantitative trait loci, with independent validation in the iPOP cohort for several signatures (Barapour et al, 2026). Together, these findings illustrate how population proteomics can move beyond describing population differences toward understanding the biological mechanisms that underlie them.

## Building the infrastructure for global population proteomics

Achieving the full potential of population proteomics will require coordinated global infrastructure. Emerging global initiatives, including π-HuB, seek to transform proof-of-principle studies into systematic population-scale resources. Whereas hPOP illustrates the value of deep cross-regional multiomics, π-HuB aims to build systematic proteome maps across populations, life stages, tissues, body fluids, and health states (π-HuB Consortium, 2024). Together with national biobanks, disease cohorts, and proteogenomic consortia, such initiatives can support population-aware reference maps, longitudinal disease trajectories, biomarker recalibration, and drug–target prioritization.

Broadening population proteomics means expanding not only the populations studied, but also the biological information captured. One direction is to deepen the measurement of the protein layer itself, moving beyond abundance to post-translational modifications, proteoforms, protein or immune complexes, extracellular-vesicle-associated proteins, and cell-type-resolved protein states. These measurements provide complementary information on how proteins are expressed, modified, assembled, transported, and compartmentalized across populations and disease states.

A second, complementary direction is to map immune recognition of the proteome. Autoantibodies are not merely another protein-abundance readout; they represent a humoral immune memory layer through which self-antigens, tissue injury, inflammation, and immune memory become detectable in blood.

Immune proteomics already illustrates the value of this expanded perspective. Autoantibody reactome profiling in relapsing polychondritis, for example, identified diagnostic autoantibodies, molecular subtypes, and activity-associated immune signatures, illustrating how immune proteomics can support diagnosis and stratification beyond conventional abundance assays (Liu et al, 2025). These findings underscore the need for a Human Autoantibody Atlas, an emerging population-aware reference framework for mapping disease-associated and context-dependent autoantibody repertoires across diseases, ancestries, environments, and life stages. The Human Autoantibody Reactome Project (HARP) Initiative, launched at the 2025 HUPO Congress in Canada, provides a timely international framework for developing this atlas as an open, population-aware, and disease-spanning effort rather than as a completed standard. By extending population proteomics from protein abundance to immune recognition, such an atlas could provide a foundation for immune biomarker discovery, molecular disease classification, longitudinal monitoring, and therapy-response stratification.

## Opportunities and challenges

Several priorities follow. First, population proteomics must broaden both geographical and ancestral representation while ensuring sufficient cohort size to enable robust within-population analyses, particularly among African, Indigenous, Latin American, Middle Eastern, South Asian, Southeast Asian, and admixed populations. Second, cross-sectional maps should be complemented by longitudinal sampling before and after migration, dietary transition, infection, medication exposure, pregnancy, aging, and disease onset. Third, integrating population-based cohorts with disease-enriched cohorts will be essential to connect molecular variation observed in healthy populations with disease mechanisms, progression trajectories, and therapeutic responses. Population-based cohorts provide reference maps of molecular diversity and identify early signatures associated with future disease risk, whereas disease-enriched cohorts enable deeper investigation of disease-specific molecular changes, clinical heterogeneity, and treatment responses. Together, these complementary resources can transform population proteomics from population-level association studies into clinically actionable frameworks for prevention, diagnosis, and precision therapy.

Fourth, equitable translation will require shared standards. Population-aware reference ranges, platform harmonization, calibration across laboratories, consent for cross-border data sharing, ethical governance, and inclusive clinical validation will determine whether the field improves equity or simply creates larger, biased databases. A recent standardized framework for circulating blood proteomics argues for technology-agnostic reference samples, donor-derived and synthetic plasma references, sample-to-reference ratios, and cross-platform quality control as practical foundations for comparable and clinically useful data (Cai et al, 2025). This is not a technical footnote; it is a prerequisite for translation.

Finally, population proteomics must avoid biological essentialism. Population-associated molecular differences should be interpreted as context-dependent biological signals shaped by genetics, environment, social exposure, microbiome ecology, health systems, and study design. The aim is not to create new categories of biological difference, but to make precision medicine generalizable, calibrated, and fair.

Population proteomics is evolving from single-region discovery toward a global science capable of supporting equitable precision medicine. hPOP provides a timely case study because it treats ancestry, geography, and relocation as central biological questions. Yet the field extends beyond any single study: large biobanks provide scale, cross-ancestry studies expose portability limits, disease cohorts drive translation, and coordinated international infrastructure will enable systematic population-scale proteomics. The central message is that precision medicine cannot remain precise for only a subset of the world’s populations. To become equitable, it must capture the protein-level biology through which human diversity becomes clinically actionable.
